# Adjuvant chemotherapy combined with pembrolizumab immunotherapy for primary triple-negative neuroendocrine carcinoma of the breast: a case report and literature review

**DOI:** 10.3389/fimmu.2026.1848635

**Published:** 2026-06-04

**Authors:** Xin Shen, Jiangnan Yang, Yang Du, Deyuan Fu

**Affiliations:** 1 Department of Thyroid and Breast Surgery, Clinical Medical College, Yangzhou University, Yangzhou, Jiangsu, China; 2 Key Laboratory of Translational Cancer Stem Cell Research, Department of Pathophysiology, Hunan Normal University Health Science Center, Changsha, China; 3 Department of Thyroid and Breast Surgery, Northern Jiangsu People’s Hospital affiliated to Yangzhou University, Yangzhou, Jiangsu, China

**Keywords:** adjuvant therapy, clinical prognosis, disease, immunohistochemistry, primary breast neuroendocrine carcinoma

## Abstract

Neuroendocrine breast cancer (NEBC) is an exceedingly rare malignant tumor, with primary triple-negative NEBC representing an even rarer and more aggressive subtype lacking standardized treatment guidelines. Although pembrolizumab combined with chemotherapy has become the standard of care for high-risk early-stage triple-negative breast cancer (TNBC) following the KEYNOTE-522 trial and recent expert consensus recommendations, its role in the rare histologic subtype of primary NEBC remains largely unexplored. We report a 71-year-old postmenopausal woman who presented with a left breast mass discovered over three years earlier. Comprehensive preoperative staging excluded extra-mammary neuroendocrine primaries. Histopathology and immunohistochemistry confirmed primary NEBC (ER−, PR−, HER2−, CgA+, Syn+, CD56+, Ki-67 index 30%), PD-L1 positivity (CPS ≥10) and germline BRCA1/2 mutation. The patient underwent curative mastectomy with sentinel lymph node biopsy (pT2N0M0, stage IIA). Adjuvant therapy comprised paclitaxel (175 mg/m²) plus cisplatin (75 mg/m²) every 3 weeks for six cycles, combined with pembrolizumab (200 mg every 3 weeks). The regimen was well tolerated with only grade 1–2 toxicities. At 12-month follow-up, the patient remains disease-free with no evidence of recurrence. To our knowledge, this is one of the first reported cases of pembrolizumab combined with platinum-based chemotherapy in the adjuvant setting for primary triple-negative NEBC. This case provides hypothesis-generating evidence for chemo-immunotherapy in this rare, high-grade histologic subtype.

## Introduction

NEBC is a rare subtype of breast cancer. Neuroendocrine tumors (NENs) can occur in multiple parts of the body, most commonly in the gastrointestinal tract, followed by the lung and breast. NEBC accounts for <1% of all breast tumors, approximately 0.1% of breast cancer, and <1% of NENs (1). The morphological features resemble those of neuroendocrine tumors that arise from the lung or gastrointestinal system. The tumor cells exhibit a prominent expression of neuroendocrine markers, including chromogranin A (CgA) and synaptophysin (Syn) (2). Given that normal breast tissue does not show the presence of neuroendocrine cells, it is presently accepted that NEBC does not arise from existing neuroendocrine cells, but might instead stem from the initial differentiation of tumor cells in breast cancer (3). Since its first report, the definition, classification, and diagnostic criteria of NEBC have undergone significant evolution, but there is still a lack of accurate and unified diagnostic criteria. Furthermore, there is overlap in diagnostic criteria with invasive breast cancer with neuroendocrine differentiation, leading to a lack of comparability in treatment and prognosis-related studies.

NEBC lacks unique clinical manifestations and imaging features, leading to insufficient clinical understanding. Currently, the treatment of NEBC mainly follows standard breast cancer treatment protocols. This article reports a case of primary NEBC and reviews and discusses relevant literature to improve clinicians’ understanding of this disease.

## Case presentation

**Figure 1:**
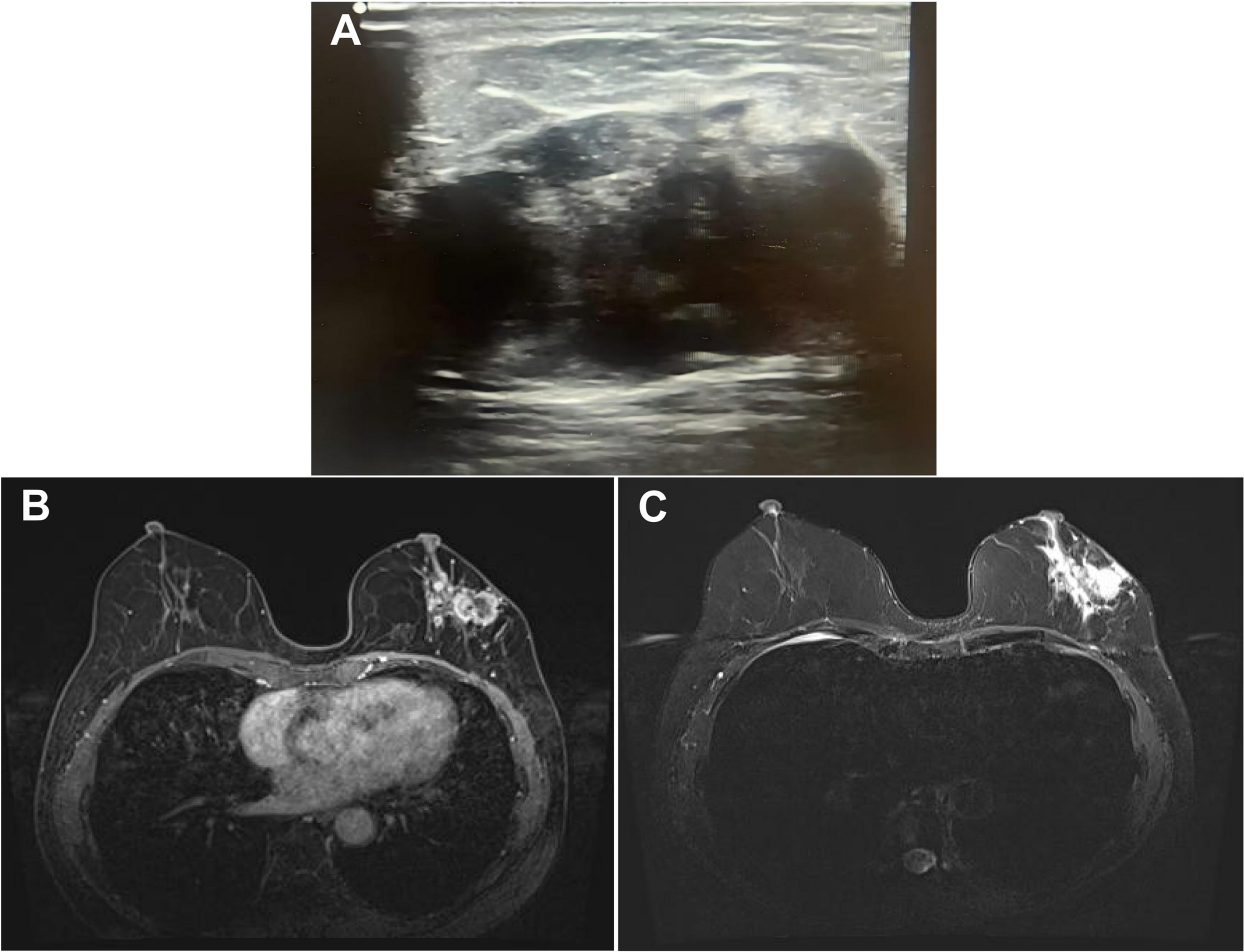
Preoperative breast imaging examination. **(A)** Ultrasound examination of the left breast showed a hypoechoic mass at the 2–3 o’clock position, measuring approximately 35×32×22 mm. Some areas showed extremely low echogenicity, with irregular edges, indistinct borders, and irregular shape. The internal echoes were chaotic and heterogeneous, and multiple microcalcifications were visible. CDFI showed linear blood flow within the mass, with a high-resistance blood flow signal (BI-RADS 4C). **(B, C)** Preoperative breast MRI examination results. Enhanced MRI scan showed an irregular, lobulated mass in the left breast with indistinct borders and infiltrative growth. The mass showed significant heterogeneous enhancement: the peripheral solid components and some areas showed obvious high signal enhancement, while a large patchy low signal area was present in the center. Multiple large and tortuous blood vessels with rich blood supply were visible inside and around the mass.

**Figure 2:**
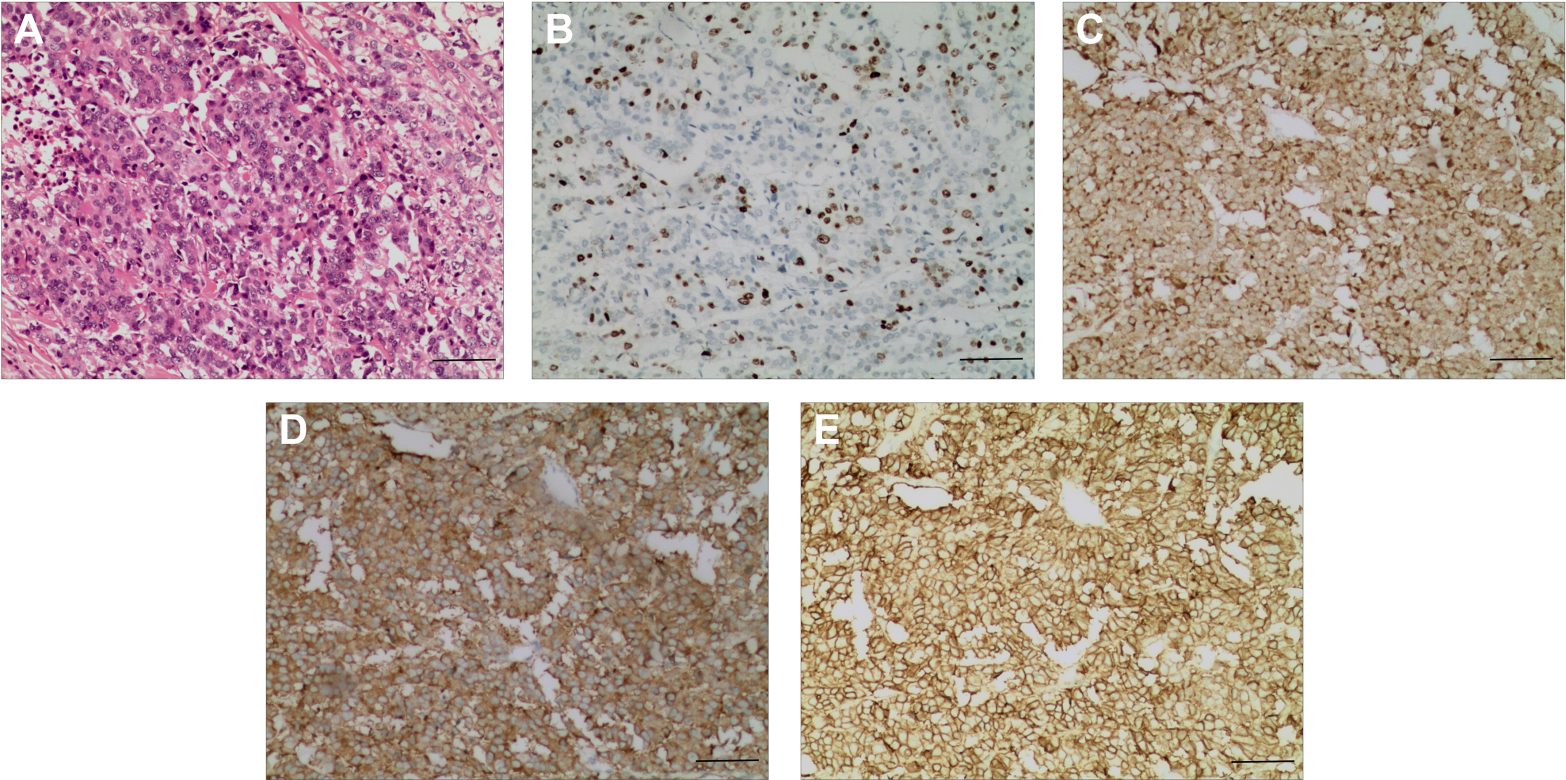
Pathological HE staining and immunohistochemical results. **(A)** NEBC pathological map (HE, ×100); **(B–E)** immunohistochemical map,**(B)** Ki-67 (+30%) ×100, **(C)** CgA(+) ×100, **(D)** Syn (+) ×100, Figure E:CD56 (+) ×100.

A 71-year-old female, postmenopausal at age 50, presented to the hospital on February 24, 2024, with a history of a left breast mass for over 3 years. Physical examination upon admission revealed symmetrical breasts, no ptosis, no skin redness or swelling, and no nipple retraction or discharge. Palpation revealed a mass approximately 3.5cm × 3cm in size, hard and homogeneous, with indistinct borders, an irregular surface, poor mobility, and no tenderness, palpable at the 3 o’clock position in the outer quadrant of the left breast, 1cm from the nipple. No axillary lymph node enlargement was observed. Breast ultrasound showed a hypoechoic mass measuring approximately 35×32×22 mm in the 2–3 o’clock position of the left breast. The mass had indistinct borders, irregular shape, and heterogeneous internal echoes with multiple microcalcifications. Strip-like blood flow was detected within the mass, and the spectrum showed high-resistance blood flow signal (BI-RADS 4C) (**Figure 1A**). Enhanced breast MRI showed a lesion in the left breast, classified as 5 by BI-RADS, accompanied by multiple abnormal enhancing lesions around the lesion, behind the nipple, and at the anterior border of the intercostal muscles in the lower quadrant (**Figures 1B, C**). To exclude extra-mammary primary neuroendocrine tumors (particularly those originating from the lung or gastrointestinal tract), the patient underwent comprehensive staging investigations prior to surgery. These included contrast-enhanced computed tomography (CT) of the chest, abdomen, and pelvis. No suspicious lesions suggestive of an alternative primary site were identified. Following a thorough preoperative evaluation, the patient underwent radical mastectomy for left breast cancer on March 11, 2024, along with a sentinel lymph node biopsy of the axilla. Postoperative pathological immunohistochemistry revealed left breast neuroendocrine carcinoma. Immunohistochemistry showed estrogen receptor (ER, -), progesterone receptor (PR, -), human epidermal growth factor receptor-2 (HER2, -), CgA(+), Syn(+), neuroadhesion molecule antibody (CD56, +), and tumor proliferation index (Ki-67,index was 30%), PD-L1 positivity (combined positive score [CPS] ≥10) and germline BRCA1/2 mutation was identified through genetic testing. As shown in **Figure 2**. The final pathological stage was T2N0M0 stage IIA.

The patient recovered smoothly post-surgery, with the incision healing primarily. Given the tumor’s triple-negative status (ER-, PR-, HER2-), Ki-67 index was 30%, PD-L1 positivity and germline BRCA1/2 mutation, indicating a high degree of malignancy, and pathological staging of pT2N0M0 (Stage IIA), a multidisciplinary consultation decided to administer adjuvant chemotherapy combined with immunotherapy. The chemotherapy regimen consisted of paclitaxel combined with cisplatin, specifically paclitaxel 175 mg/m² intravenously on day 1 and cisplatin 75 mg/m² intravenously on day 1, with each cycle lasting 21 days, for a planned total of 6 cycles. Simultaneously, immunotherapy was administered in combination with pembrolizumab 200 mg, intravenously every 3 weeks. The patient tolerated the treatment well, with the main adverse reactions being grade I-II myelosuppression, mild gastrointestinal reactions, and peripheral nerve numbness, all of which were effectively controlled with symptomatic and supportive treatment. No grade 3–4 serious adverse events occurred, and the entire treatment plan was successfully completed.

Postoperative follow-up was standardized, including clinical physical examination, left breast and regional lymph node ultrasound, enhanced CT scan of the chest and abdomen every 3 months, and serum tumor marker testing. The patient underwent surgery on March 11, 2024. As of March 2025 (12 months postoperatively), all follow-up results showed no local recurrence or distant metastasis of the tumor. The patient was in good general condition with no significant discomfort, and the disease-free survival has now reached 12 months. The patient remains disease-free at 12 months, representing a promising short-term outcome in this rare tumor type.

Consent was secured from the patient or their immediate family members. This research adheres to the pertinent stipulations of the Declaration of Helsinki established by the World Medical Association.

## Discussion

### The concept and classification of NEBC

Neuroendocrine tumors usually occur in the nervous and endocrine systems, including pancreatic tumors, paragangliomas, pheochromocytomas, etc. (4), but reports on NEBC are extremely rare, and there is still no consensus on the diagnostic criteria for NEBC. Feyrter et al. (5) first proposed the concept of NEBC in 1963, pointing out that the microscopic morphology of NEBC is similar to that of intestinal carcinoids. In 2003, WHO categorized neuroendocrine carcinoma as a distinct subtype of breast cancer (6). This classification is described as follows: after ruling out primary tumors located outside the breast, particularly those originating in the lungs and gastrointestinal tract, it is determined that over 50% of the tumor cells demonstrate the expression of immunohistochemical neuroendocrine markers necessary for diagnosis. In 2012, WHO classified breast neuroendocrine tumors into three subtypes: Poorly differentiated neuroendocrine carcinomas or small cell carcinomas, well-differentiated neuroendocrine tumors and invasive breast cancer (7). In 2019, the WHO classified breast neuroendocrine tumors into two major categories: neuroendocrine tumors and neuroendocrine carcinomas. It also proposed mixed carcinomas, including invasive carcinomas and neuroendocrine carcinomas (8).

### Clinical manifestations, imaging, and pathological diagnosis of NEBC

NEBC mostly occurs in middle-aged and elderly, postmenopausal women aged 43 to 70 years (9), and they often seek medical attention due to the accidental discovery of a breast lump. The most common initial manifestation of NEBC is a single breast mass, while a small number of cases present with local redness and swelling of the breast skin and nipple discharge (10).

The imaging features of NEBC patients are not specific and are not significantly different from other types of breast malignancies. Analogous to the common luminal subtype of breast cancer, NEBC has the potential to spread to various locations, frequently affecting the bones and liver (11, 12). Some scholars have pointed out that NEBC appears as a local mass on mammography, usually without microcalcifications (13); on ultrasound, it appears as an irregularly shaped hypoechoic mass with indistinct borders, calcifications within the mass, and relatively rich blood flow signals (14). There are currently few reported cases of NEBC on MRI.

Currently, there are few reports on large clinical samples of MRI findings of NEBC. It is mostly characterized by a unilateral mass with clear borders and shallow lobulation, and T2WI shows mainly heterogeneous high signal (15). Consistent with these findings, preoperative ultrasound in our case classified the lesion as BI-RADS 4C, while enhanced MRI showed a BI-RADS 5 mass with multiple surrounding abnormal enhancing foci, yet final staging confirmed no lymph node or distant metastasis. The lack of characteristic features in imaging examinations cannot confirm NEBC, and pathology is required for a definitive diagnosis. The histogenesis of NEBC is still unclear. Several studies indicate that it arises from the transformation of neuroendocrine cells found in the breast tissue. More studies suggest that NEBC is the neuroendocrine differentiation of epithelial cancer stem cells (16). Most NEBCs are classified as high-grade histologically. Most patients are already in the advanced stage when they are diagnosed. The rate of metastasis to regional lymph nodes can reach approximately 40%, while the rate of distant metastasis is around 20% (17). The current diagnosis of NEBC is mainly based on immunohistochemistry by core needle biopsy (18). NEBC is similar to sarcoma, showing infiltrative or expansive growth. When there is bleeding and mucus, its texture is soft and has a gelatinous appearance. Under the microscope, NEBCs are composed of round or spindle-shaped cells with palisade-like changes in their nuclei and a large number of eosinophilic granules in their cytoplasm (19). Several markers such as CgA, Syn, and neuron-specific enolase (NSE) are present in neuroendocrine cells and show positive silver staining.

### Treatment options for NEBC

NEBC cases are rare, and there are no clear treatment guidelines at home and abroad. However, surgery remains the primary option for initial treatment and is considered the main therapeutic approach. The adjuvant therapy after surgery is determined according to the size, stage, grade and receptor status of the patient’s tumor (18). Radiotherapy, chemotherapy, targeted therapy and endocrine therapy are adjuvant treatment methods. At present, there is a lack of trustworthy information regarding the involvement of adjuvant and neoadjuvant therapies in NEBC. Consequently, the same criteria are applied as those for other forms of invasive breast cancer. For breast cancer that is positive for ER and PR, endocrine therapy plays a crucial role in treatment (20). The combination of CDK-4/6 inhibitors with aromatase inhibitors can enhance the prognosis for patients with breast cancer who are at a high risk of recurrence. Chemotherapy can be used for high-risk patients with hormone receptor positivity and high Ki-67 expression (21). In the present case, the patient was a 71-year-old postmenopausal woman with triple-negative (ER-/PR-/HER2-) NEBC, Ki-67 index was 30%, and stage IIA (pT2N0M0,stage IIA). Following multidisciplinary discussion and considering the tumor’s triple-negative status, high Ki-67 index (30%), PD-L1 positivity, germline BRCA1/2 mutation, and stage IIA disease, adjuvant chemotherapy with paclitaxel (175 mg/m²) plus cisplatin (75 mg/m²) every 3 weeks for 6 cycles combined with pembrolizumab (200 mg every 3 weeks) was selected. This regimen was specifically chosen because: (1) platinum agents such as cisplatin exhibit enhanced activity in BRCA-mutated TNBC through synthetic lethality and immunogenic cell death; (2) pembrolizumab is supported by KEYNOTE-522 data and expert consensus for PD-L1-positive high-risk early TNBC; (3) the combination avoids anthracycline-related cardiotoxicity in an elderly patient; and (4) the high-grade NEBC histology further favors platinum-based therapy. The treatment was well tolerated with only grade 1–2 toxicities.

### Immunotherapy for NEBC

To place our case in the context of existing evidence, we conducted a systematic literature search in the PubMed database from January 1, 2008, to December 31, 2025. The search strategy combined the following terms: (“breast” OR “mammary”) AND (“neuroendocrine carcinoma” OR “neuroendocrine tumor” OR “neuroendocrine neoplasm” OR “carcinoid” OR “NEBC” OR “primary neuroendocrine breast”). We included only English-language case reports and small case series that described histopathologically confirmed primary breast neuroendocrine carcinoma with documented immunohistochemical expression of at least one neuroendocrine marker (chromogranin A, synaptophysin, and/or CD56). Exclusion criteria were: review articles without new original cases, metastatic or secondary neuroendocrine carcinomas of the breast, publications in languages other than English, and cases lacking sufficient clinical, immunohistochemical, treatment, or outcome details. Two authors independently screened titles and abstracts, followed by full-text review; disagreements were resolved by consensus. This process yielded 19 eligible cases, which are summarized in **Table 1**. Among them, Janosky et al. (22) used an anthracycline-containing regimen for neoadjuvant chemotherapy in NEBC patients, and the results showed that 50% of tumor cells in the pathological specimens were reduced after neoadjuvant chemotherapy, and the patients subsequently underwent successful breast-conserving surgery. Marijanović et al. (23) reported on a case of HER2-positive NEBC patient who, after receiving systemic therapy including combined anti-HER2 therapy, achieved distant recurrence-free survival for up to 9 years. Currently, no targeted therapies are available for patients with NEBC who are negative for ER and PR and exhibit low levels of Her-2 expression. Notably, our case adds to this limited evidence base. Unlike the HER2-positive case reported by Marijanović et al. with long-term survival after anti-HER2 therapy, our triple-negative, high-grade NEBC patient achieved a favorable short-term outcome (12-month disease-free survival) with platinum-based chemotherapy plus immunotherapy, suggesting that immune checkpoint inhibitors may warrant further exploration in this molecular subtype.

**Table 1:** NEBC-related case report summary.

Case number	Year	Gender/age	ER	PR	HER2	Systemic treatment	Outcome	Reference
1	2020	Female/50	Pos	Pos	Neg	Chemo + ET	No evidence of recurrence at 44 months	(9)
2	2019	Female/69	Pos	Pos	Neg	Chemo (FEC + docetaxel) + ET (anastrozole)	Disease-free for 8 years, then liver + lymph node metastases	(29)
3	2018	Female/73	Pos	Pos	Neg	ET	Disease-free at 4 years	(30)
4	2017	Female/65	Neg	Neg	Neg	Chemo + RT (but systemic: Chemo)	Good clinical response on PET-CT at 3 months; no further follow-up	(31)
5	2017	Female/57	Pos	Pos	n/a	Chemo	Alive with stable bone metastases at 18 months	(32)
6	2018	Female/32	Pos	Pos	Pos	Neo-adjuvant Chemo + RT	No long-term outcome reported	(33)
7	2016	Female/42	Neg	Neg	Neg	(Systemic: none specified)	Disease-free >36 months after surgery	(34)
8	2016	Female/42	Pos	Pos	Neg	Chemo + ET	No evidence of metastasis at 1-year follow-up	(35)
9	2015	Female/34	Pos	Pos	Neg	Refused adjuvant therapy	Disease-free at 4 years post-surgery	(36)
10	2015	Female/34	Neg	Neg	Neg	Neo-adjuvant anthracycline-based Chemo (+ further Chemo)	Rapid local recurrence and systemic metastasis (lungs + bones) despite multimodal therapy	(22)
11	2015	Female/62	Pos	Pos	Neg	Chemo + ET	No symptoms after completion of adjuvant therapy	(37)
12	2014	Female/51	Pos	Pos	Neg	Chemo + ET	Lymph node recurrence at 10 years; disease-free 6 months after excision + letrozole	(38)
13	2012	Female/46	n/a	n/a	n/a	Adjuvant Chemo (Cisplatin,Epirubicin,Etoposid)	No recurrence at 6 months post-surgery	(39)
14	2012	Female/44	Pos	Pos	Neg	Adjuvant Chemo (FEC)	Disease-free at 48 months	(40)
15	2012	Female/63	Neg	Neg	Neg	Adjuvant Chemo (Uracil + Tegafur)	Disease-free at 44 months	(41)
16	2009	Female/28	Neg	Pos	n/a	Neo-adjuvant Chemo (Cisplatin/Etoposide)	Complete remission at 12 months	(42)
17	2008	Female/27	n/a	n/a	n/a	Adjuvant Chemo	No recurrence or metastasis at 18 months	(43)
18	2024	Female/59	Pos	Neg	Neg	Adjuvant Chemo (AC ×4) + ET	No recurrence reported	(44)
19	2025	Female/73 (series)	Mixed	Mixed	Mixed	Pertuzumab + Trastuzumab + Paclitaxel → Anastrozole (HER2+ cases); ET for luminal cases	Case series of 6 NEBC (4 LCNEC+2 SCNEC); 83% developed distant metastases within 4 years	(45)

The KEYNOTE-522 clinical trial found that pembrolizumab in combination with chemotherapy has become the standard of care for high-risk early-stage TNBC, and it received FDA approval in 2021 (24). However, the triple-negative subtype of NEBC represents a unique and extremely rare histological entity with distinctive biology, aggressive behavior, and limited subtype-specific data. We conducted a systematic review of 19 published cases of primary breast neuroendocrine carcinoma (2008–2025) and found no prior reports on the use of PD-1/PD-L1 inhibitors as adjuvant therapy in triple-negative NEBC. Most cases of triple-negative NEBC in the literature were treated with chemotherapy alone, showing variable or relatively aggressive outcomes. Although there have been recent isolated reports of immunotherapy for advanced or small cell NEBC, there is no description of the combination of paclitaxel, cisplatin, and pembrolizumab for early-stage high-risk TNBC. This case, demonstrating 12 months of disease-free survival with this chemoimmunotherapy regimen in pathologically confirmed primary TN-NEBC, provides novel hypothesis-generating evidence for the potential application of PD-1 blockade in this rare histological subtype. This observation highlights the potential synergistic effect of platinum-based drugs and anti-PD-1 therapy in high Ki-67 NEBC and warrants validation in future prospective, biomarker-driven trials.

Meanwhile, related clinical trials have also reported that pembrolizumab combined with chemotherapy significantly improved pathological complete response rate and event-free survival. The patient’s tumor expressed PD-L1 (CPS≥10) and carried BRCA1/2 germline mutations; these two key biomarkers are associated with enhanced immunogenicity and greater benefit from PD-1 blockade and platinum-based chemotherapy. Platinum-based drugs such as cisplatin can promote immunogenic cell death, enhance tumor antigen presentation, and synergize with PD-1 inhibitors, thereby enhancing anti-tumor immunity. The specific immune mechanism is as follows: Platinum-based drugs such as cisplatin induce immunogenic cell death (ICD) by triggering the release of damage-associated molecular patterns (DAMPs), including calreticulin, high-mobility group box 1 (HMGB1), and adenosine triphosphate (ATP). This process promotes dendritic cell maturation, antigen cross-presentation, and recruitment of tumor-infiltrating lymphocytes, thereby creating a more inflammatory tumor microenvironment, which synergizes with PD-1 inhibitors to enhance the anti-tumor immune response (25, 26).

Although specific data on immunotherapy for NEBC remain extremely limited, high-grade neuroendocrine tumors typically exhibit higher Ki-67 indices and may be more immunogenic than well-differentiated counterparts, while PD-L1 expression varies among extramammary neuroendocrine carcinomas. This case, a triple-negative, high Ki-67 NEBC patient with PD-L1 positivity and BRCA1/2 mutations, provided a strong biological basis for adding pembrolizumab to the paclitaxel-cisplatin regimen, achieving encouraging short-term efficacy. This case underscores the importance of comprehensive biomarker analysis for rare histological subtypes in guiding personalized chemotherapy-immunotherapy.

Beyond the established role of chemo-immunotherapy exemplified by the KEYNOTE-522 trial, several novel treatment strategies are emerging for primary triple-negative NEBC (3, 20). Given the germline BRCA1/2 mutation identified in this case and the triple-negative phenotype of this primary NEBC, PARP inhibitors (e.g., olaparib) represent a promising targeted option, supported by the OlympiA trial data in BRCA-mutated high-risk HER2-negative breast cancer (27) as well as emerging evidence suggesting utility of PARP inhibitors in breast neuroendocrine neoplasms (20). Antibody-drug conjugates (ADCs) such as sacituzumab govitecan have also shown encouraging activity in high-grade neuroendocrine carcinomas of the breast, including small-cell subtypes (28), offering a potential future avenue even in the adjuvant or metastatic setting. In addition, peptide receptor radionuclide therapy (PRRT) with radiolabeled somatostatin analogs may be considered in somatostatin receptor-positive NEBC cases, while DLL3-targeted T-cell engagers are under investigation for high-grade neuroendocrine carcinomas (3). These biomarker-driven approaches, combined with comprehensive genomic profiling, highlight the potential to further personalize therapy for this rare and aggressive histologic subtype and warrant evaluation in prospective studies (19, 20).

## Conclusion

In conclusion, this case illustrates the promising short-term use of adjuvant chemo-immunotherapy (paclitaxel, cisplatin, and pembrolizumab) in primary triple-negative NEBC, a rare and aggressive histologic subtype. It adds hypothesis-generating evidence to the sparse literature by demonstrating a 12-month disease-free survival in the adjuvant setting. However, the relatively short follow-up duration precludes definitive conclusions regarding long-term efficacy or superiority over standard regimens. Larger prospective studies with extended follow-up and appropriate biomarker assessment are essential to establish optimal treatment strategies for this uncommon entity.

## Data Availability

The original contributions presented in the study are included in the article/Supplementary Material. Further inquiries can be directed to the corresponding authors.
